# PPDIST, global 0.1° daily and 3-hourly precipitation probability distribution climatologies for 1979–2018

**DOI:** 10.1038/s41597-020-00631-x

**Published:** 2020-09-11

**Authors:** Hylke E. Beck, Seth Westra, Jackson Tan, Florian Pappenberger, George J. Huffman, Tim R. McVicar, Gaby J. Gründemann, Noemi Vergopolan, Hayley J. Fowler, Elizabeth Lewis, Koen Verbist, Eric F. Wood

**Affiliations:** 1 Department of Civil and Environmental Engineering, Princeton University, Princeton, New Jersey USA; 2 School of Civil, Environmental and Mining Engineering, University of Adelaide, Adelaide, Australia; 3 Universities Space Research Association, Columbia, Maryland USA; 4 NASA Goddard Space Flight Center, Greenbelt, Maryland USA; 5 Forecast Department, European Centre for Medium-Range Weather Forecasts (ECMWF), Reading, UK; 6 Mesoscale Atmospheric Processes Laboratory, NASA Goddard Space Flight Center, Greenbelt, Maryland USA; 7 CSIRO Land and Water, Black Mountain, Canberra Australia; 8 Australian Research Council Centre of Excellence for Climate Extremes, Canberra, Australia; 9 Delft University of Technology, Water Management, Delft, Netherlands; 10 School of Engineering, Newcastle University, New castle upon Tyne, UK; 11 UNESCO International Hydrological Programme, 7, Place de Fontenoy, 75352 Paris, France

**Keywords:** Hydrology, Climate sciences

## Abstract

We introduce the Precipitation Probability DISTribution (PPDIST) dataset, a collection of global high-resolution (0.1°) observation-based climatologies (1979–2018) of the occurrence and peak intensity of precipitation (*P*) at daily and 3-hourly time-scales. The climatologies were produced using neural networks trained with daily *P* observations from 93,138 gauges and hourly *P* observations (resampled to 3-hourly) from 11,881 gauges worldwide. Mean validation coefficient of determination (*R*^2^) values ranged from 0.76 to 0.80 for the daily *P* occurrence indices, and from 0.44 to 0.84 for the daily peak *P* intensity indices. The neural networks performed significantly better than current state-of-the-art reanalysis (ERA5) and satellite (IMERG) products for all *P* indices. Using a 0.1 mm 3 h^−1^ threshold, *P* was estimated to occur 12.2%, 7.4%, and 14.3% of the time, on average, over the global, land, and ocean domains, respectively. The highest *P* intensities were found over parts of Central America, India, and Southeast Asia, along the western equatorial coast of Africa, and in the intertropical convergence zone. The PPDIST dataset is available via www.gloh2o.org/ppdist.

## Abstract (summary)

**Table:** 

Measurement(s)	hydrological precipitation process • precipitation occurrence • precipitation intensity
Technology Type(s)	Neural networks models • Gauge or Meter Device • satellite imaging of a planet • reanalyses
Factor Type(s)	daily and 3-hourly time-scales • year of data collection
Sample Characteristic - Environment	climate system
Sample Characteristic - Location	Earth (planet)

Machine-accessible metadata file describing the reported data: 10.6084/m9.figshare.12815585

## Background & Summary

Knowledge about the climatological probability distribution of precipitation (*P*) is essential for a wide range of scientific, engineering, and operational applications^1–3^. In the scientific realm, *P* distributional information is used, for example, to evaluate *P* outputs from climate models (e.g., Dai^4^ and Bosilovich *et al*.^5^) and to reduce systematic biases in gridded *P* datasets (e.g., Zhu and Luo^6^, Xie *et al*.^7^, and Karbalaee *et al*.^8^). In the engineering sector, information on the upper tail of the *P* distribution is crucial for planning, design, and management of infrastructure (e.g., Lumbroso *et al*.^9^ and Yan *et al*.^10^). In an operational context, reliable information on the *P* distribution can be used to improve flood and drought forecasting systems (e.g., Cloke and Pappenberger^11^, Hirpa *et al*.^12^, and Siegmund *et al*.^13^).

Past studies deriving (quasi-)global climatologies of *P* distributional characteristics typically relied on just one of the three main sources of *P* data (satellites, reanalyses, or gauges), each with strengths and weaknesses:

1. Ricko *et al*.^14^ used the TMPA satellite *P* product^15^ while Trenberth and Zhang^16^ and Li *et al*.^17^ used the CMORPH satellite *P* product^7,18^ (both available from 2000 onwards) to estimate climatological *P* characteristics. Satellite *P* estimates have several advantages, including a high spatial (~0.1°) and temporal (≤3-hourly) resolution, and near-global coverage (typically <60°N/S)^19,20^. However, satellite *P* retrieval is confounded by complex terrain^21–23^ and surface snow and ice^22,24^, and there are major challenges associated with the detection of snowfall^25,26^.
2. Courty *et al*.^27^ used *P* estimates between 1979 and 2018 from the ERA5 reanalysis^28^ to derive probability distribution parameters of annual *P* maxima for the entire globe. Reanalyses assimilate vast amounts of *in situ* and satellite observations into numerical weather prediction models^29^ to produce a temporally and spatially consistent continuous record of the state of the atmosphere, ocean, and land surface. However, reanalyses are affected by deficiencies in model structure and parameterization, uncertainties in observation error statistics, and historical changes in observing systems^5,30,31^.
3. Sun *et al*.^32^ calculated *P* occurrence estimates directly from gauge observations, while Dietzsch *et al*.^33^ used the gauge-based interpolated GPCC dataset^34^ to estimate *P* characteristics for the entire land surface. Gauges provide accurate estimates of *P* at point scales and therefore have been widely used to evaluate satellite- and reanalysis-based *P* products (e.g., Hirpa *et al*.^35^, Zambrano-Bigiarini *et al*.^36^, and Beck *et al*.^37^). However, only 16% of the global land surface (excluding Antarctica) has a gauge located at <25-km distance^38,39^, gauge placement is topographically biased towards low elevations^40,41^, and there has been a significant decline in the number of active gauges over recent decades^42^. Additionally, interpolation of gauge observations generally leads to systematic overestimation of low intensity events and underestimation of high intensity events^43,44^.

**Fig. 1:**
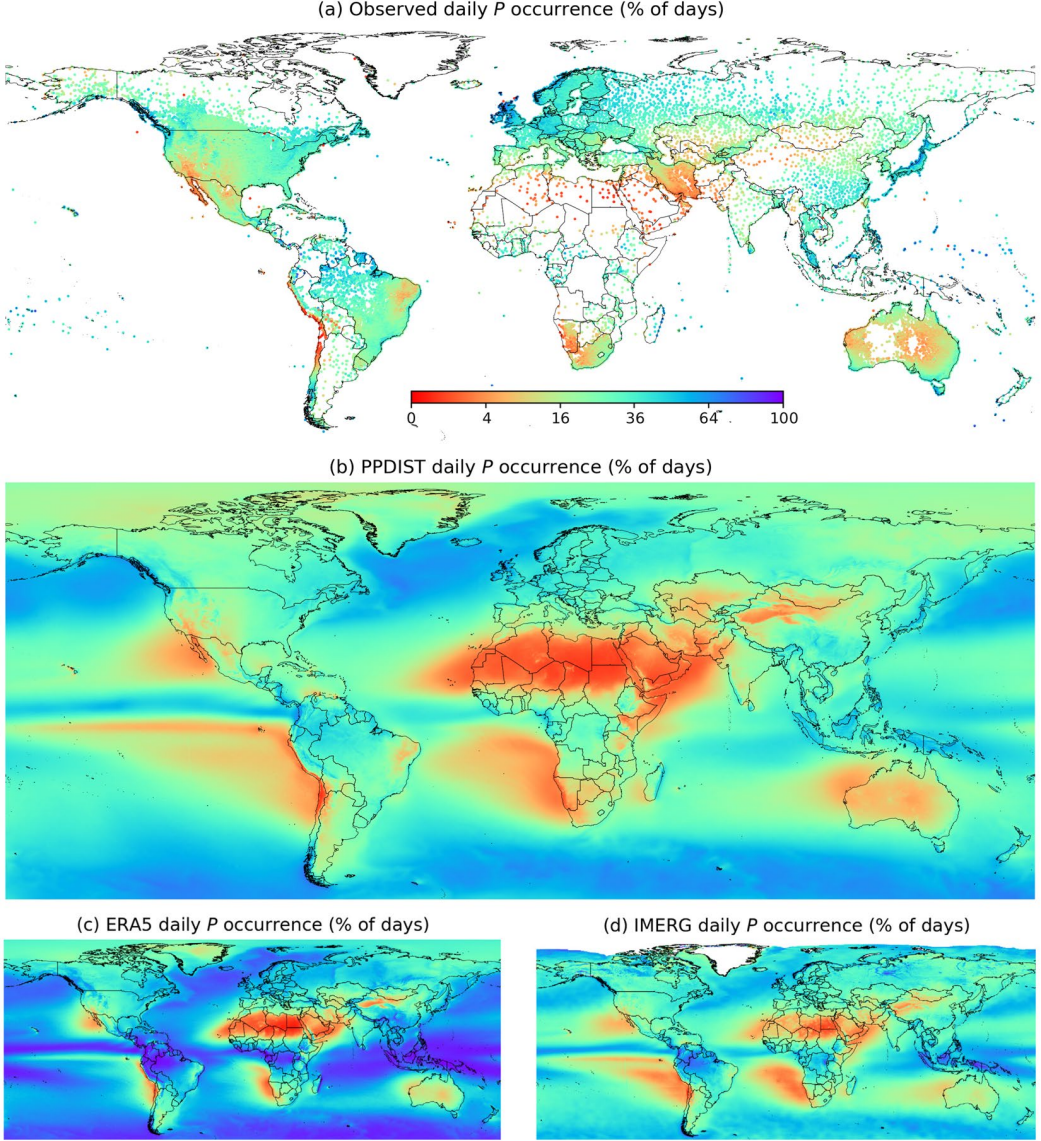
The >0.5 mm d^−1^ daily *P* occurrence according to (**a**) the gauge observations, (**b**) the PPDIST dataset, (**c**) the ERA5 reanalysis, and (**d**) the IMERG satellite product. Supplement Fig. S2 presents an equivalent figure for the >0.1 mm 3 h^−1^ 3-hourly *P* occurrence. The other PPDIST *P* occurrence indices can be viewed by accessing the dataset.

**Fig. 2:**
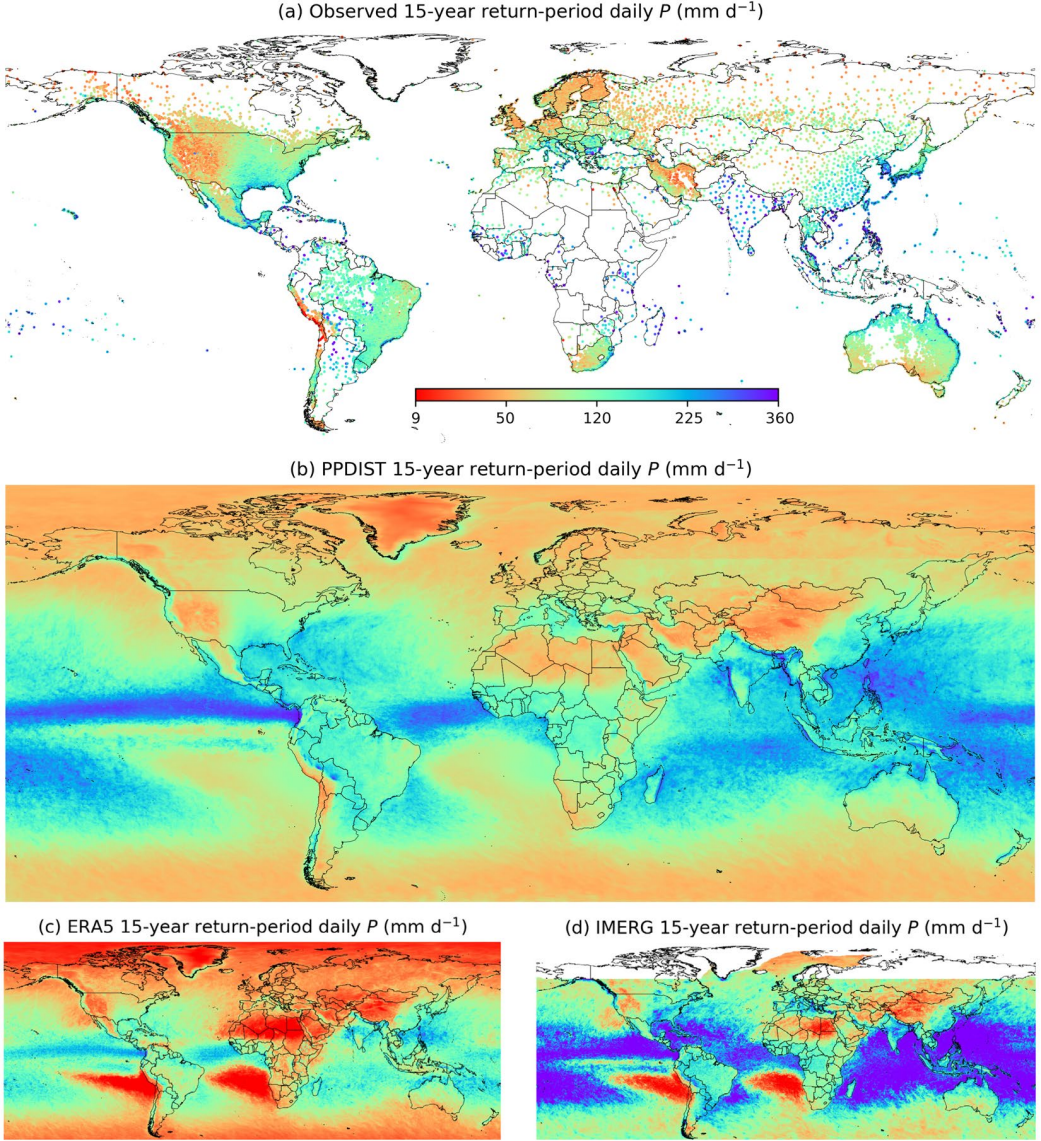
The 15-year return-period daily *P* intensity according to (**a**) the gauge observations, (**b**) the PPDIST dataset, (**c**) the ERA5 reanalysis, and (**d**) the IMERG satellite product. IMERG has data gaps at high latitudes (>60°N/S) precluding the calculation of the 15-year return-period daily *P* intensity. Supplement Fig. S3 presents an equivalent figure for the 15-year return-period 3-hourly *P* intensity. The other PPDIST *P* intensity indices can be viewed by accessing the dataset.

**Fig. 3:**
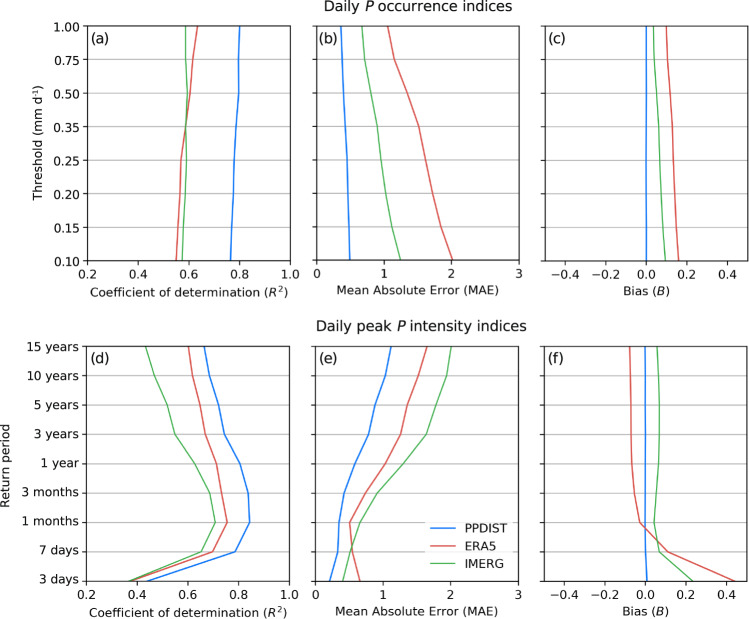
Performance of the PPDIST dataset, the ERA5 reanalysis, and the IMERG satellite product in estimating the daily *P* occurrence and peak *P* intensity indices. For the PPDIST dataset, we calculated the mean of the ten validation scores (one for each cross-validation iteration). The training scores were not shown as they were nearly identical to the validation scores. All scores were calculated using square-root-transformed observed and estimated values. Supplement Fig. S4 presents an equivalent figure for the 3-hourly *P* indices.

**Fig. 4:**
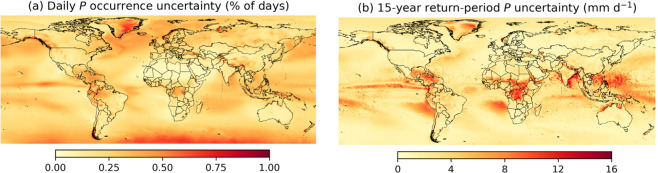
PPDIST uncertainty estimates for (**a**) the >0.5 mm d^−1^ daily *P* occurrence and (**b**) the 15-year return-period daily *P* intensity. The uncertainty represents the spread of the ten cross-validation iterations. Supplement Fig. S5 presents an equivalent figure for the >0.1 mm 3 h^−1^ *P* occurrence and the 15-year return-period 3-hourly *P* intensity.

Here, we introduce the Precipitation Probability DISTribution (PPDIST) dataset, a collection of high-resolution (0.1°) fully global climatologies (1979–2018) of eight *P* occurrence indices (using different thresholds to discriminate between *P* and no-*P*) and nine peak *P* intensity indices (using different return periods) at daily and 3-hourly time-scales (Figs. 1b and 2b). The climatologies were produced using neural network ensembles trained to estimate the *P* indices using quality-controlled *P* observations from an unprecedented database consisting of 93,138 daily and 11,881 hourly gauges worldwide (Fig. 1a and Supplement Fig. S2a). Ten global *P*-, climate-, and topography-related predictors derived from all three main *P* data sources — satellites, reanalyses, and gauges — were used as input to the neural networks to enhance the accuracy of the estimates (Table 1). Ten-fold cross-validation was used to obtain an ensemble of neural networks, evaluate the generalizability of the approach, and quantify the uncertainty. The performance was assessed using the coefficient of determination (*R*^2^), the mean absolute error (MAE), and the bias (*B*; Fig. 3). The neural networks yielded mean validation *R*^2^ values (calculated using the validation subsets of observations) of 0.78 and 0.86 for the daily and 3-hourly *P* occurrence indices, respectively, and 0.72 and 0.83 for the daily and 3-hourly peak *P* intensity indices, respectively (Fig. 3 and Supplement Fig. S4). The neural networks outperformed current state-of-the-art reanalysis (ERA5^28^) and satellite (IMERG^45,46^) products for all *P* indices and performance metrics (Fig. 3). ERA5 consistently overestimated the *P* occurrence (Fig. 1c), while IMERG tended to overestimate the peak *P* intensity (Fig. 2d). The uncertainty in the neural network-based estimates of the *P* indices was quantified globally and was generally higher in sparsely gauged, mountainous, and tropical regions (Fig. 4). Overall, these results suggest that the neural networks provide a useful high-resolution, global-scale picture of the *P* probability distribution. It should be noted that although the climatologies are provided as gridded maps, the estimates represent the point scale rather than the grid-box scale, due to the use of gauge observations for the training^43,47^.

**Table 1:** Predictors used in the neural networks to estimate the *P* occurrence and peak *P* intensity indices.

Predictor	Description (units	Resolution	Data source	Used for daily *P* indices	Used for 3-hourly *P* indices
CAPE	Convective available potential energy (J kg^−1^)	0.28°	ERA5-HRES reanalysis (mean over 1979–2018)^28^ (https://cds.climate.copernicus.eu)	✓	✓
CldCov	Cloud cover frequency (%)	1 km	Wilson *et al*.^114^ (www.earthenv.org/cloud); gaps over ocean filled using linear interpolation	✓	✓
Elev	Square-root transformed surface elevation smoothed using 10-km filter (—)	90 m	MERIT^115^ (http://hydro.iis.u-tokyo.ac.jp/~;yamadai/MERIT_DEM/) over land; 0 over ocean	✓	✓
MAP1^a^	Square-root-transformed mean annual precipitation (—) from WorldClim V2	1 km (land); 0.28° (ocean)	WorldClim V2^84^ (www.worldclim.org) over land; ERA5^28^ (https://cds.climate.copernicus.eu) over ocean	✓	✓
MAP2^a^	Difference in square-root-transformed mean annual precipitation (—) between WorldClim V2 and CHPclim V2	0.05° (land); 0.28° (ocean)	CHPclim V1^83^ (www.chc.ucsb.edu/data/chpclim) and WorldClim V2^84^ (www.worldclim.org) over land; 0 over ocean	✓	✓
MAT	Mean annual air temperature (°C)	1 km (land); 0.28° (ocean)	See MAP1	✓	✓
Snow Frac	Long-term fraction of total *P* falling as snow (—) calculated according to Legates and Bogart^116^	1 km (land); 0.28° (ocean)	See MAP1	✓	✓
Lat	Absolute geographical latitude (°)	0.1°	—	✓	✓
ERA5^b^	ERA5-HRES (1979–2018) reanalysis *P* occurrence (% of time) and peak *P* intensity (mm d^-1^ or mm 3 h^-1^) indices	0.28°	Hersbach *et al*.^28^ (https://cds.climate.copernicus.eu)	✓	✓
IMERG^b^	IMERG Late run (IMERGHHL) V06 (2000–2018) satellite-based *P* occurrence (% of time) and peak *P* intensity (mm d^−1^ or mm 3 h^−1^) indices	0.1°	Huffman *et al*.^45,46^ (https://pmm.nasa.gov/data-access/downloads/gpm)	✓	✓
${{\rm{PPDIST}}}_{{\rm{dly}}}^{{\rm{c}}}$	Daily PPDIST estimates of the *P* occurrence and peak *P* intensity indices	0.1°	The PPDIST dataset derived in this study (www.gloh2o.org/ppdist)	✗	✓

^a^Two mean annual *P* climatologies were used to account the uncertainty in mean annual *P* estimates.

^b^The ERA5 and IMERG predictors represent estimates of the *P* index subject of the estimation.

^c^Daily PPDIST estimates of the *P* index subject of the estimation were used as predictor for the 3-hourly estimates.

Since the eight PPDIST *P* occurrence climatologies were similar, we only present and discuss results for the >0.5 mm d^−1^ daily *P* occurrence climatology (Fig. 1b), although the others can be viewed by accessing the dataset. The *P* occurrence was particularly high (>0.5 mm d^−1^ on >50% of days) in windward coastal areas of Chile, Colombia, the British Isles, and Norway, along the Pacific coast of North America, and over parts of Southeast Asia (Fig. 1b)^16,48–51^. Conversely, the *P* occurrence was particularly low (>0.5 mm d^−1^ on <1% of days) in the Atacama and Sahara deserts. At a daily time-scale using a 0.5 mm d^−1^ threshold, *P* was found to occur 34.4%, 24.9%, and 38.9% of days, on average, over the global, land, and ocean domains, respectively (Fig. 1b). At a 3-hourly time-scale using a 0.1 mm 3 h^−1^ threshold, *P* was found to occur 12.2%, 7.4%, and 14.3% of the time, on average, over the global, land, and ocean domains, respectively (Supplement Fig. S2b). These estimates are in line with previous estimates derived from the satellite-based CMORPH product^16^ and from the satellite-, reanalysis-, and gauge-based MSWEP product^52^. The higher average *P* occurrence over ocean than over land reflects the unlimited water supply^53^. The PPDIST > 0.5 mm d^−1^ daily *P* occurrence climatology (Fig. 1b) exhibits reasonable visual agreement with similar climatologies from previous studies despite the use of different data sources, spatiotemporal resolutions, and thresholds to discriminate between *P* and no-*P* (Sun *et al*.^32^, their Fig. 1; Ellis *et al*.^54^, their Fig. 3; Trenberth and Zhang^16^, their Fig. 4; and Beck *et al*.^52^, their Fig. 8).

Since the nine PPDIST peak *P* intensity climatologies were similar, we only present and discuss results for the 15-year return-period daily *P* climatology (Fig. 2b). Peak *P* intensities were markedly higher at low latitudes (<30°N/S; Fig. 2b), due to the higher air temperatures which lead to more intense *P* in accordance with the Clausius-Clapeyron relationship^55–57^. The highest 15-year return-period *P* intensities (>200 mm d^−1^) over land were found in parts of Central America, India, and Southeast Asia, along the western equatorial coast of Africa, and over oceans in the intertropical convergence zone (Fig. 2b)^17,48,58,59^. Conversely, the lowest 15-year return-period *P* intensities (<50 mm d^−1^) were found in the desert regions of the world, at high latitudes where frontal *P* dominates, and over southeastern parts of the Atlantic and Pacific oceans where semi-permanent anticyclones prevail^54,60^. The PPDIST 15-year return-period daily *P* climatology (Fig. 2b) exhibits reasonable visual agreement with a maximum one-day *P* climatology derived by Dietzsch *et al*.^33^ (their Fig. 5d) from the gauge-based GPCC Full Data Daily dataset^34^, as well as with a 3.4-year return-period 3-hourly *P* climatology derived by Beck *et al*.^52^ (their Fig. 7) from the satellite-, reanalysis-, and gauge-based MSWEP dataset^52^.

## Methods

### Gauge observations and quality control

Our initial database of daily *P* gauge observations comprised 149,095 gauges worldwide and was compiled from (i) the Global Historical Climatology Network-Daily (GHCN-D) dataset (ftp.ncdc.noaa.gov/pub/data/ghcn/daily/)^61^ (102,954 gauges), (ii) the Global Summary Of the Day (GSOD) dataset (https://data.noaa.gov; 24,730 gauges), (iii) the Latin American Climate Assessment & Dataset (LACA&D) dataset (http://lacad.ciifen.org/; 231 gauges), (iv) the Chile Climate Data Library (www.climatedatalibrary.cl; 716 gauges), and (v) national datasets for Brazil (11,678 gauges; www.snirh.gov.br/hidroweb/apresentacao), Mexico (5398 gauges), Peru (255 gauges), and Iran (3133 gauges). Our initial database of hourly *P* gauge observations (resampled to 3-hourly) comprised 14,350 gauges from the Global Sub-Daily Rainfall (GSDR) dataset^62^ produced as part of the INTElligent use of climate models for adaptatioN to non-Stationary hydrological Extremes (INTENSE) project^63^.

Gauge data can have considerable measurement errors and therefore quality control is important^64–66^. To eliminate long series of erroneous zeros frequently present in daily GSOD records^67,68^, we calculated a central moving mean with a one-year window (assigning a value only if at least half a year of values were present), and discarded daily and 3-hourly *P* observations with a coincident moving mean of zero. To eliminate long series of *P* without zeros, indicative of biased reporting practices or quality control issues, we calculated a central moving minimum with a one-year window (assigning a value only if at least half a year of values were present), and discarded daily and 3-hourly *P* observations with a coincident moving minimum greater than zero. We only used observations between January 1st, 1979, and December 31st, 2018, and discarded gauges with (i) < 3 years of remaining data, (ii) the largest daily *P* > 2000 mm d^−1^ or 3-hourly *P* > 500 mm 3 h^−1^ (approximately the maximum recorded 24-h and 3-h *P*, respectively^69^), (iii) the highest *P* value < 2 mm d^−1^ or <1 mm 3 h^−1^, or (iv) the lowest *P* value > 1 mm d^−1^ or >0.5 mm 3 h^−1^. The resulting database comprised 93,138 gauges with daily data (Fig. 1a) and 11,881 gauges with 3-hourly data (Supplement Fig. S2a). See Supplement Fig. S1 for record lengths of the daily and 3-hourly gauge data.

### Precipitation occurrence and peak intensity indices

We considered eight *P* occurrence indices (related to the lower tail of the *P* distribution) and nine peak *P* intensity indices (related to the upper tail of the *P* distribution), all calculated for both the daily and 3-hourly time-scale. The eight *P* occurrence indices measure the percentage of time with *P* using thresholds of 0.1, 0.15, 0.2, 0.25, 0.35, 0.5, 0.75, and 1 mm d^−1^ or mm 3 h^−1^ to discriminate between *P* and no-*P*. The *P* occurrence was calculated using the percentileofscore function of the scipy Python module^70^. We considered multiple thresholds because (i) different researchers and meteorological agencies adopt different definitions of “wet day” and “rain day”, (ii) the most suitable threshold depends on the application, and (iii) the estimation accuracy depends on the threshold^16,71^.

The nine peak *P* intensity indices measure the magnitude of daily or 3-hourly *P* events for return periods of 3 days, 7 days, 1 month, 3 months, 1 year, 3 years, 5 years, 10 years, and 15 years. The magnitudes were calculated using the percentile function of the numpy Python module^72,73^. Percentiles were calculated according to *p* = 100 − 100/(365.25 · *R*) for the daily time-scale and *p* = 100 − 100/(365.25 · 8 · *R*) for the 3-hourly time-scale, where *p* is the percentile (%) and *R* is the return period (years). Gauges with <3 years of data were discarded per the preceding subsection. However, we only calculated the three highest return periods if the record lengths were longer than 5, 10, and 15 years, respectively. We did not consider return periods >15 years because (i) the number of observations would significantly decrease, and (ii) the IMERG dataset, which was used as predictor, spans only 20 years.

### Neural network ensembles

We used feed-forward artificial neural networks based on the multilayer perceptron^74^ to produce global maps of the *P* indices. Neural networks are models composed of interconnected neurons able to model complex non-linear relationships between inputs and outputs. Neural networks have been successfully applied in numerous studies to estimate different aspects of *P* (e.g., Coulibaly *et al*.^75^, Kim and Pachepsky^76^, and Nastos *et al*.^77^). We used a neural network with one hidden layer composed of 10 nodes with the logistic sigmoid activation function. The stochastic gradient descent method was used to train the weights. To make the training more efficient, the inputs (i.e., the predictors; Table 1) were standardized by subtracting the means and dividing by the standard deviations of the global predictor maps. Additionally, the outputs (i.e., the *P* indices) were square-root transformed to reduce the skewness of the distributions, and standardized using means and standard deviations of the observed *P* indices.

To obtain an ensemble of neural networks, evaluate the generalizability of the approach, and quantify the uncertainty, we trained neural networks for each *P* index using ten-fold cross-validation. For each cross-validation iteration, the observations were partitioned into subsets of 90% for training and 10% for validation. The partitioning was random, although each observation was used only once for validation. Since the validation subsets are completely independent and were not used to train the neural networks, they allow quantification of the performance at ungauged locations.

We subsequently applied the trained neural networks using global 0.1° maps of the predictors, yielding an ensemble of ten global maps for each *P* index (one for each cross-validation iteration). The values were destandardized by multiplying by the standard deviation and adding the mean of observed *P* indices, and back-transformed by squaring the values, after which we used the mean of the ensemble to provide a ‘best estimate’ and the standard deviation to provide an indication of the uncertainty. As a last step, we sorted the eight mean *P* occurrence estimates for each 0.1° grid cell to ensure that they continuously decrease as a function of the thresholds, and sorted the nine mean peak *P* intensity estimates to ensure that they continuously increase as a function of the percentiles. The uncertainty estimates were re-ordered according to the mean estimates.

### Predictors

Table 1 presents the predictors used as input to the neural networks. The predictors were upscaled from their native resolution to 0.1° using average resampling. To highlight broad-scale topographic features, the elevation predictor (Elev) was smoothed using a Gaussian kernel with a 10-km radius following earlier studies^78,79^. The Elev predictor was square-root transformed to increase the importance of topographic features at low elevation^80^. The mean annual air temperature predictor (MAT) was included because higher air temperatures are generally associated with higher *P* intensities^56,81^.

Mean annual *P* predictors (MAP1 and MAP2) were included due to the high correlations found between mean and extreme values of *P*^14,82^. To account for the uncertainty in mean annual *P* estimates, we considered two state-of-the-art climatologies: CHPclim V1^83^ and WorldClim V2^84^. Since the use of correlated predictors can increase training time^85^, we did not directly use both climatologies as separate predictors. Instead, we used square-root transformed mean annual *P* from WorldClim as predictor MAP1, and the difference in square-root transformed mean annual *P* between CHPclim and WorldClim as predictor MAP2. The square-root transformation was used to increase the importance of smaller values.

The ERA5 and IMERG predictors represent maps of the *P* index subject of the estimation derived from ERA5 and IMERG *P* data, respectively. The ERA5 and IMERG predictors were added because they provide valuable independent information about the *P* distribution with (near-)global coverage. All predictors cover the entire land surface, with the exception of IMERG, which was often not available at latitudes higher than approximately 60°N/S^46^. This issue was addressed by replacing missing IMERG estimates of the *P* indices with equivalent estimates from ERA5, resulting in a duplication of ERA5 at high latitudes. *P* distributional characteristics associated with different durations often exhibit similar spatial patterns^27,86^. To allow the 3-hourly estimates to take advantage of the much better availability of daily *P* observations (Figs. 1a and 2a), we used the daily climatology of the *P* index subject of the estimation as additional predictor for producing the 3-hourly climatology.

### Performance metrics

We assessed the performance of the PPDIST dataset, the ERA5 reanalysis, and the IMERG satellite product in estimating the *P* indices using the gauge observations as reference. Prior to the assessment, the *P* indices of the products and the reference were square-root transformed to reduce the skewness of the distributions. For the PPDIST dataset, we calculated the average of the ten scores calculated using the ten independent validation subsets of observations (one for each cross-validation iteration). For ERA5 and IMERG, we simply calculated the scores using all available observations. The following three performance metrics were used:

1. The coefficient of determination (*R*^2^), which measures the proportion of variance accounted for^87^, ranges from 0 to 1, and has its optimum at 1.

2. The mean absolute error (MAE), calculated as:

1 $${\rm{MAE}}=\frac{\mathop{\sum }\limits_{i=1}^{n}\left|{X}_{i}^{e}-{X}_{i}^{o}\right|}{n},$$

where ${X}_{i}^{e}$ and ${X}_{i}^{o}$ represent estimated and observed values of the transformed *P* indices, respectively, *i* = 1, …, *n* denotes the observations, a*n*d *n* is the sample size. The MAE ranges from 0 to ∞ and has its optimum at 0. We used the MAE, instead of the more common root mean square error (RMSE), because the errors are unlikely to follow a normal distribution^88,89^.

3. The bias (*B*), defined as:

2 $$B=\frac{\mathop{\sum }\limits_{i=1}^{n}{X}_{i}^{e}-{X}_{i}^{o}}{\mathop{\sum }\limits_{i=1}^{n}{X}_{i}^{e}-{X}_{i}^{o}}.$$

*B* ranges from −1 to 1 and has its optimum at 0. Other widely used bias formulations, which contain the sum of only the observations in the denominator rather than the sum of both the observations and estimates^90,91^, were not used, because they yield unwieldy high *B* values when the observations tend to zero.

## Data Records

The PPDIST dataset is available for download at figshare^92^ and www.gloh2o.org/ppdist. The data are provided as a zip file containing four netCDF-4 files (www.unidata.ucar.edu/software/netcdf) with gridded observations, mean estimates, and uncertainty estimates of (i) the daily *P* occurrence (daily_occurrence_point_scale.nc), (ii) the daily peak *P* intensity (daily_intensity_point_scale.nc), (iii) the 3-hourly *P* occurrence (3hourly_occurrence_point_scale.nc), and (iv) the 3-hourly peak *P* intensity (3hourly_intensity_point_scale.nc). The grids have a 0.1° spatial resolution and are referenced to the World Geodetic Reference System 1984 (WGS 84) ellipsoid. The observational grids were produced by averaging the observations if multiple observations were present in a single grid cell. The netCDF-4 files can be viewed, edited, and analyzed using most Geographic Information Systems (GIS) software packages, including ArcGIS, QGIS, and GRASS.

## Technical Validation

Figure 3 presents the performance of the PPDIST dataset, the ERA5 reanalysis, and the IMERG satellite product in estimating the daily *P* indices (Supplement Fig. S4 presents the performance in estimating the 3-hourly *P* indices). The PPDIST scores represent averages calculated from the ten independent validation subsets of observations (one for each cross-validation iteration). The PPDIST *R*^2^ values ranged from 0.76 to 0.80 (mean 0.78) for the daily *P* occurrence indices, 0.85 to 0.88 (mean 0.86) for the 3-hourly *P* occurrence indices, 0.44 to 0.84 (mean 0.72) for the daily peak *P* intensity indices, and 0.72 to 0.88 (mean 0.83) for the 3-hourly peak *P* intensity indices (Fig. 3 and Supplement Fig. S4). The 3-hourly *R*^2^ values were thus higher than the daily *R*^2^ values, likely because the 3-hourly gauge observations tend to be of higher quality^62^. The markedly lower *R*^2^ for the 3-day return-period daily *P* intensity reflects the fact that the majority (58%) of the observations were 0 mm d^−1^. For the ≥3-year return-period daily and 3-hourly *P* indices, the lower performance reflects the uncertainty associated with estimating the magnitude of heavy *P* events^93–95^. The PPDIST scores were superior to the ERA5 and IMERG scores for every *P* index and performance metric by a substantial margin. The PPDIST *B* scores were close to 0 for all *P* indices, indicating that the estimates do not exhibit systematic biases, apart from those already present in the gauge data due to wind-induced under-catch^64,96^ and the underreporting of light *P* events^65^. Overall, these results suggest that the PPDIST dataset provides a reliable global-scale picture of the *P* probability distribution.

The ERA5 and IMERG products yielded mean *R*^2^ values of 0.59 and 0.58 for the daily *P* occurrence indices, 0.69 and 0.49 for the 3-hourly *P* occurrence indices, 0.65 and 0.56 for the daily *P* intensity indices, and 0.79 and 0.57 for the 3-hourly *P* intensity indices, respectively (Fig. 3 and Supplement Fig. S4). The global patterns of *P* occurrence and peak *P* intensity were thus generally better estimated by ERA5 than by IMERG. However, ERA5 consistently overestimated the *P* occurrence and the lowest return periods, in line with evaluations of other climate models^30,37,97^. Additionally, ERA5 generally showed lower values for the highest return periods (Fig. 3), which is at least partly due to the spatial scale mismatch between point-scale observations and grid-box averages^43,47,98,99^. Conversely, ERA5 strongly overestimated the *P* intensity over several grid cells in eastern Africa in particular (Fig. 2c), likely due to the presence of so-called “rain bombs” caused by numerical artifacts^100^.

The overly high *P* occurrence of IMERG in the tropics (Fig. 1d) may be at least partly attributable to the inclusion of relatively noisy *P* estimates from the SAPHIR sensor on board the tropical-orbiting Megha-Tropiques satellite^46^. Similar to the TMPA 3B42 and CMORPH satellite products, IMERG overestimated the *P* occurrence over several large rivers (notably the Amazon, the Lena, and the Ob), likely due to misinterpretation of the emissivity of small inland water bodies as *P* by the passive microwave *P* retrieval algorithms^101^. The too low *P* occurrence at high latitudes reflects the challenges associated with the retrieval of snowfall^26,102^ and light *P*^103,104^. Furthermore, IMERG tended to underestimate the *P* intensity over regions of complex topography (e.g., over Japan and the west coast of India; Fig. 2d), which is a known limitation of the product^23,105^. The too high *P* intensity in the south central US may stem from the way *P* estimates from the various passive microwave sensors are intercalibrated to the radar-based CORRA product in the IMERG algorithm^46^.

## Usage Notes

The PPDIST dataset will be useful for numerous purposes, such as the evaluation of *P* from climate models (e.g., Dai^4^ and Bosilovich *et al*^5^.), the distributional correction of gridded *P* datasets (e.g., Zhu and Luo^6^, Xie *et al*.^7^, and Karbalaee *et al*.^8^), the design of infrastructure in poorly gauged regions (e.g., Lumbroso *et al*.^9^), and in hydrological modelling where *P* intensity is required (e.g., Donohue *et al*.^106^ and Liu *et al*.^107^). However, some caveats should be kept in mind:

1. The uncertainty estimates included in the PPDIST dataset provide an indication of the reliability of the climatologies. The uncertainty is generally higher in sparsely gauged, mountainous, and tropical regions (Fig. 4). The uncertainty estimates may, however, be on the low end, because while we accounted for uncertainty in the mean annual *P* predictor, we did not account for uncertainty in the other predictors.
2. The *P* occurrence estimates using low thresholds to discriminate between *P* and no-*P* (≤0.25 mm d^−1^ or mm 3 h^−1^) should be interpreted with caution due to the detection limits of gauges (~0.25 mm for the US National Weather Service [NWS] 8” standard gauge^108^) and satellite sensors (~0.8 mm h^−1^ for the TRMM microwave imager [TMI]^109^ and possibly higher for other sensors with coarser footprints) and the underreporting of light *P* events^65^.
3. *P* observations from unshielded gauges (e.g., the US NWS 8” standard gauge) are subject to wind-induced gauge under-catch and therefore underestimate rainfall by 5–15% and snowfall by 20–80%^64,96^. This underestimation is reflected in the PPDIST estimates. The Precipitation Bias CORrection (PBCOR) dataset^110^ (www.gloh2o.org/pbcor) provides a means to ameliorate this bias to some degree.
4. The PPDIST climatologies were derived from gauge observations and therefore represent the point scale. Compared to point-scale estimates, grid-box averages tend to exhibit more frequent *P* of lower intensity^16,43,47,99^. Point-scale *P* occurrence estimates can be transformed to grid-box averages using, for example, the equations of Osborn and Hulme^111^, whereas the peak *P* intensity estimates can be transformed to grid-box averages using, for example, the areal reduction factors collated by Pietersen *et al*.^112^.
5. As mentioned before, ERA5 strongly overestimates the *P* intensity over several grid cells in eastern Africa in particular (Fig. 2c). Since ERA5 was used as predictor, these artifacts are also present, albeit less pronounced, in the PPDIST *P* intensity estimates (Fig. 2b).
6. Some of the PPDIST climatologies (e.g., the 15-year return-period daily *P* climatology; Fig. 2b) exhibit a longitudinal discontinuity at 60°N/S, reflecting the spatial extent of the IMERG data (Table 1). Additionally, some of the climatologies exhibit discontinuities at coastlines (e.g., along the west coast of Patagonia), due to the use of different data sources for land and ocean for the MAP1, MAP2, MAT, and SnowFrac predictors.

## Supplementary information

Supplementary Information

## Data Availability

The neural networks used to produce the PPDIST dataset were implemented using the MLPRegressor class of the scikit-learn Python module^113^. The *P* occurrence for different thresholds was calculated using the percentileofscore function of the scipy Python module^70^, whereas the *P* magnitudes for different return periods were calculated using the percentile function of the numpy Python module^72,73^. The other codes are available upon request from the first author. The predictor, IMERG, and ERA5 data are available via the URLs listed in Table 1. Most of the gauge observations are available via the URLs provided in the “Gauge observations and quality control” subsection. Part of the GSDR database and some of the national databases are only available upon request.

## References

[1] Tapiador FJ (2012). Global precipitation measurement: Methods, datasets and applications. Atmospheric Research.

[2] Kucera PA (2013). Precipitation from space: Advancing Earth system science. Bulletin of the American Meteorological Society.

[3] Kirschbaum DB (2017). NASA’s remotely sensed precipitation: A reservoir for applications users. Bulletin of the American Meteorological Society.

[4] Dai A (2006). Precipitation characteristics in eighteen coupled climate models. Journal of Climate.

[5] Bosilovich MG, Chen J, Robertson FR, Adler RF (2008). Evaluation of global precipitation in reanalyses. Journal of Applied Meteorology and Climatology.

[6] Zhu Y, Luo Y (2015). Precipitation calibration based on the frequency-matching method. Weather and Forecasting.

[7] Xie P (2017). Reprocessed, bias-corrected CMORPH global high-resolution precipitation estimates from 1998. Journal of Hydrometeorology.

[8] Karbalaee N, Hsu K, Sorooshian S, Braithwaite D (2017). Bias adjustment of infrared based rainfall estimation using passive microwave satellite rainfall data. Journal of Geophysical Research: Atmospheres.

[9] Lumbroso DM, Boyce S, Bast H, Walmsley N (2011). The challenges of developing rainfall intensity-duration-frequency curves and national flood hazard maps for the Caribbean. Journal of Flood Risk Management.

[10] Yan H (2019). Next-generation intensity-duration-frequency curves to reduce errors in peak flood design. Journal of Hydrologic Engineering.

[11] Cloke HL, Pappenberger F (2009). Ensemble flood forecasting: A review. Journal of Hydrology.

[12] Hirpa FA (2016). The effect of reference climatology on global flood forecasting. Journal of Hydrometeorology.

[13] Siegmund J, Bliefernicht J, Laux P, Kunstmann H (2015). Toward a seasonal precipitation prediction system for West Africa: Performance of CFSv2 and high-resolution dynamical downscaling. Journal of Geophysical Research: Atmospheres.

[14] Ricko M, Adler RF, Huffman GJ (2016). Climatology and interannual variability of quasi-global intense precipitation using satellite observations. Journal of Climate.

[15] Huffman GJ (2007). The TRMM Multisatellite Precipitation Analysis (TMPA): quasi-global, multiyear, combined-sensor precipitation estimates at fine scales. Journal of Hydrometeorology.

[16] Trenberth KE, Zhang Y (2018). How often does it really rain?. Bulletin of the American Meteorological Society.

[17] Li, X.-F. *et al*. Global distribution of the intensity and frequency of hourly precipitation and their responses to ENSO. *Climate Dynamics* 1–17 (2020).

[18] Joyce RJ, Janowiak JE, Arkin PA, Xi P (2004). CMORPH: A method that produces global precipitation estimates from passive microwave and infrared data at high spatial and temporal resolution. Journal of Hydrometeorology.

[19] Stephens GL, Kummerow CD (2007). The remote sensing of clouds and precipitation from space: a review. Journal of the Atmospheric Sciences.

[20] Sun Q (2018). A review of global precipitation datasets: data sources, estimation, and intercomparisons. Reviews of Geophysics.

[21] Prakash S (2018). A preliminary assessment of GPM-based multi-satellite precipitation estimates over a monsoon dominated region. Journal of Hydrology.

[22] Cao Q, Painter TH, Currier WR, Lundquist JD, Lettenmaier DP (2018). Estimation of precipitation over the OLYMPEX domain during winter 2015/16. Journal of Hydrometeorology.

[23] Beck HE (2019). Daily evaluation of 26 precipitation datasets using Stage-IV gauge-radar data for the CONUS. Hydrology and Earth System Sciences.

[24] Kidd C (2012). Intercomparison of high-resolution precipitation products over northwest Europe. Journal of Hydrometeorology.

[25] Levizzani V, Laviola S, Cattani E (2011). Detection and measurement of snowfall from space. Remote Sensing.

[26] Skofronick-Jackson G (2015). Global Precipitation Measurement Cold Season Precipitation Experiment (GCPEX): for measurement’s sake, let it snow. Bulletin of the American Meteorological Society.

[27] Courty LG, Wilby RL, Hillier JK, Slater LJ (2019). Intensity-duration-frequency curves at the global scale. Environmental Research Letters.

[28] Hersbach, H. *et al*. The ERA5 global reanalysis. *Quarterly Journal of the Royal Meteorological Society* (2020).

[29] Bauer P, Thorpe A, Brunet G (2015). The quiet revolution of numerical weather prediction. Nature.

[30] Stephens, G. L. *et al*. Dreary state of precipitation in global models. *Journal of Geophysical Research: Atmospheres* **115** (2010).

[31] Kang S, Ahn J-B (2015). Global energy and water balances in the latest reanalyses. Asia-Pacific Journal of Atmospheric Sciences.

[32] Sun Y, Solomon S, Dai A, Portmann RW (2006). How often does it rain?. Journal of Climate.

[33] Dietzsch, F. *et al*. A global ETCCDI-based precipitation climatology from satellite and rain gauge measurements. *Climate* **5** (2017).

[34] Schamm K (2014). Global gridded precipitation over land: a description of the new GPCC First Guess Daily product. Earth System Science Data.

[35] Hirpa FA, Gebremichael M, Hopson T (2010). Evaluation of high-resolution satellite precipitation products over very complex terrain in Ethiopia. Journal of Applied Meteorology and Climatology.

[36] Zambrano-Bigiarini M, Nauditt A, Birkel C, Verbist K, Ribbe L (2017). Temporal and spatial evaluation of satellite-based rainfall estimates across the complex topographical and climatic gradients of Chile. Hydrology and Earth System Sciences.

[37] Beck HE (2017). Global-scale evaluation of 22 precipitation datasets using gauge observations and hydrological modeling. Hydrology and Earth System Sciences.

[38] Beck HE (2017). MSWEP: 3-hourly 0.25° global gridded precipitation (1979–2015) by merging gauge, satellite, and reanalysis data. Hydrology and Earth System Sciences.

[39] Kidd C (2017). So, how much of the Earth’s surface is covered by rain gauges?. Bulletin of the American Meteorological Society.

[40] Briggs PR, Cogley JG (1996). Topographic bias in mesoscale precipitation networks. Journal of Climate.

[41] Schneider U (2014). GPCC’s new land surface precipitation climatology based on quality-controlled *in situ* data and its role in quantifying the global water cycle. Theoretical and Applied Climatology.

[42] Mishra, A. K. & Coulibaly, P. Developments in hydrometric network design: A review. *Reviews of Geophysics* **47** (2009).

[43] Ensor LA, Robeson SM (2008). Statistical characteristics of daily precipitation: comparisons of gridded and point datasets. Journal of Applied Meteorology and Climatology.

[44] Hofstra, N., Haylock, M., New, M. & Jones, P. D. Testing E-OBS European high-resolution gridded data set of daily precipitation and surface temperature. *Journal of Geophysical Research: Atmospheres* **114** (2009).

[45] Huffman, G. J. *et al*. NASA global Precipitation Measurement (GPM) Integrated Multi-satellitE Retrievals for GPM (IMERG). Algorithm Theoretical Basis Document (ATBD), NASA/GSFC, Greenbelt, MD 20771, USA (2014).

[46] Huffman, G. J., Bolvin, D. T. & Nelkin, E. J. Integrated Multi-satellitE Retrievals for GPM (IMERG) technical documentation. Tech. Rep., NASA/GSFC, Greenbelt, MD 20771, USA (2018).

[47] Olsson J, Berg P, Kawamura A (2015). Impact of RCM spatial resolution on the reproduction of local, subdaily precipitation. Journal of Hydrometeorology.

[48] Dai A (2001). Global precipitation and thunderstorm frequencies. Part I: Seasonal and interannual variations. Journal of Climate.

[49] Qian J-H (2008). Why precipitation is mostly concentrated over islands in the Maritime Continent. Journal of the Atmospheric Sciences.

[50] Ogino S-Y, Yamanaka MD, Mori S, Matsumoto J (2016). How much is the precipitation amount over the tropical coastal region?. Journal of Climate.

[51] Curtis S (2019). Means and long-term trends of global coastal zone precipitation. Scientific Reports.

[52] Beck HE (2019). MSWEP V2 global 3-hourly 0.1° precipitation: methodology and quantitative assessment. Bulletin of the American Meteorological Society.

[53] Schlosser CA, Houser PR (2007). Assessing a satellite-era perspective of the global water cycle. Journal of Climate.

[54] Ellis, T. D., L’Ecuyer, T., Haynes, J. M. & Stephens, G. L. How often does it rain over the global oceans? The perspective from CloudSat. *Geophysical Research Letters* **36** (2009).

[55] Hardwick Jones, R., Westra, S. & Sharma, A. Observed relationships between extreme sub-daily precipitation, surface temperature, and relative humidity. *Geophysical Research Letters* **37** (2010).

[56] Peleg N (2018). Intensification of convective rain cells at warmer temperatures observed from high-resolution weather radar data. Journal of Hydrometeorology.

[57] Allan, R. P. *et al*. Advances in understanding large-scale responses of the water cycle to climate change. *Annals of the New York Academy of Sciences* (2020). 10.1111/nyas.14337 32246848

[58] Zipser EJ, Cecil DJ, Liu C, Nesbitt SW, Yorty DP (2006). Where are the most intense thunderstorms on earth?. Bulletin of the American Meteorological Society.

[59] Liu C, Zipser EJ (2015). The global distribution of largest, deepest, and most intense precipitation systems. Geophysical Research Letters.

[60] Behrangi A, Tian Y, Lambrigtsen BH, Stephens GL (2014). What does CloudSat reveal about global land precipitation detection by other spaceborne sensors?. Water Resources Research.

[61] Menne MJ, Durre I, Vose RS, Gleason BE, Houston TG (2012). An overview of the Global Historical Climatology Network-Daily database. Journal of Atmospheric and Oceanic Technology.

[62] Lewis E (2019). GSDR: a global sub-daily rainfall dataset. Journal of Climate.

[63] Blenkinsop S (2018). The INTENSE project: using observations and models to understand the past, present and future of sub-daily rainfall extremes. Advances in Science and Research.

[64] Goodison, B. E., Louie, P. Y. T. & Yang, D. WMO solid precipitation intercomparison. Tech. Rep. WMO/TD-872, World Meteorological Organization, Geneva (1998).

[65] Daly C, Gibson WP, Taylor GH, Doggett MK, Smith JI (2007). Observer bias in daily precipitation measurements at United States cooperative network stations. Bulletin of the American Meteorological Society.

[66] Sevruk B, Ondrás M, Chvíla B (2009). The WMO precipitation measurement intercomparisons. Atmospheric Research.

[67] Durre I, Menne MJ, Gleason BE, Houston TG, Vose RS (2010). Comprehensive automated quality assurance of daily surface observations. Journal of Applied Meteorology and Climatology.

[68] Funk C (2015). The climate hazards infrared precipitation with stations—a new environmental record for monitoring extremes. Scientific Data.

[69] Meteorological Organization (WMO), W. *Guide to hydrological practices, volume II: Management of water resources and applications of hydrological practices*, http://www.wmo.int/pages/prog/hwrp/publications/guide/english/168_Vol_II_en.pdf (WMO, Geneva, Switzerland, 2009).

[70] Virtanen P (2020). SciPy 1.0: Fundamental algorithms for scientific computing in Python. Nature Methods.

[71] Haylock, M. R. *et al*. A European daily high-resolution gridded data set of surface temperature and precipitation for 1950–2006. *Journal of Geophysical Research: Atmospheres* **113** (2008).

[72] Oliphant, T. E. NumPy: A guide to NumPy. USA: Trelgol Publishing. www.numpy.org (2006).

[73] van der Walt S, Colbert SC, Varoquaux G (2011). The Numpy array: A structure for efficient numerical computation. Computing in Science Engineering.

[74] Bishop CM (1995). Neural networks for pattern recognition.

[75] Coulibaly P, Dibike YB, Anctil F (2005). Downscaling precipitation and temperature with temporal neural networks. Journal of Hydrometeorology.

[76] Kim J-W, Pachepsky YA (2010). Reconstructing missing daily precipitation data using regression trees and artificial neural networks for SWAT streamflow simulation. Journal of Hydrology.

[77] Nastos P, Paliatsos A, Koukouletsos K, Larissi I, Moustris K (2014). Artificial neural networks modeling for forecasting the maximum daily total precipitation at Athens, Greece. Atmospheric Research.

[78] Hutchinson MF (1998). Interpolation of rainfall data with thin plate smoothing splines — part I: Two dimensional smoothing of data with short range correlation. Journal of Geographic Information and Decision Analysis.

[79] Smith RB (2003). Orographic precipitation and air mass transformation: An Alpine example. Quarterly Journal of the Royal Meteorological Society.

[80] Roe GH (2005). Orographic precipitation. Annual Review of Earth and Planetary Sciences.

[81] Molnar P, Fatichi S, Gaál L, Szolgay J, Burlando P (2015). Storm type effects on super Clausius-Clapeyron scaling of intense rainstorm properties with air temperature. Hydrology and Earth System Sciences.

[82] Benestad R, Nychka D, Mearns L (2012). Spatially and temporally consistent prediction of heavy precipitation from mean values. Nature Climate Change.

[83] Funk C (2015). A global satellite assisted precipitation climatology. Earth System Science Data.

[84] Fick SE, Hijmans RJ (2017). WorldClim 2: new 1-km spatial resolution climate surfaces for global land areas. International Journal of Climatology.

[85] Halkjær, S. & Winther, O. The effect of correlated input data on the dynamics of learning. In *NIPS*, 169–175, http://papers.nips.cc/paper/1254-the-effect-of-correlated-input-data-on-the-dynamics-of-learning (1996).

[86] Overeem A, Buishand A, Holleman I (2008). Rainfall depth-duration-frequency curves and their uncertainties. Journal of Hydrology.

[87] Rao, C. R. *Linear statistical inference and its applications* (2 edn, John Wiley and Sons, New York, 1973).

[88] Chai T, Draxler RR (2014). Root mean square error (RMSE) or mean absolute error (MAE)? – Arguments against avoiding RMSE in the literature. Geoscientific Model Development.

[89] Willmott, C. J., Robeson, S. M. & Matsuura, K. Climate and other models may be more accurate than reported. *Eos* **98** (2017).

[90] Sharifi, E., Steinacker, R. & Saghafian, B. Assessment of GPM-IMERG and other precipitation products against gauge data under different topographic and climatic conditions in Iran: preliminary results. *Remote Sensing* **8** (2016).

[91] Gupta HV, Kling H, Yilmaz KK, Martinez GF (2009). Decomposition of the mean squared error and NSE performance criteria: Implications for improving hydrological modelling. Journal of Hydrology.

[92] Beck HE (2020). figshare.

[93] Zolina O, Kapala A, Simmer C, Gulev SK (2004). Analysis of extreme precipitation over Europe from different reanalyses: a comparative assessment. Global and Planetary Change.

[94] Mehran A, AghaKouchak A, Capabilities A (2014). of satellite precipitation datasets to estimate heavy precipitation rates at different temporal accumulations. Hydrological Processes.

[95] Herold N, Behrangi A, Alexander LV (2017). Large uncertainties in observed daily precipitation extremes over land. Journal of Geophysical Research: Atmospheres.

[96] Legates, D. R. *A climatology of global precipitation*. Ph.D. thesis, University of Delaware (1988).

[97] Herold N, Alexander LV, Donat MG, Contractor S, Becker A (2016). How much does it rain over land?. Geophysical Research Letters.

[98] Tustison B, Harris D, Foufoula-Georgiou E (2001). Scale issues in verification of precipitation forecasts. Journal of Geophysical Research: Atmospheres.

[99] Chen C-T, Knutson T (2008). On the verification and comparison of extreme rainfall indices from climate models. Journal of Climate.

[100] Harrigan S (2020). GloFAS-ERA5 operational global river discharge reanalysis 1979–present. Earth System Science Data Discussions.

[101] Tian, Y. & Peters-Lidard, C. D. Systematic anomalies over inland water bodies in satellite-based precipitation estimates. *Geophysical Research Letters* **34** (2007).

[102] You Y, Wang N-Y, Ferraro R, Rudlosky S (2017). Quantifying the snowfall detection performance of the GPM microwave imager channels over land. Journal of Hydrometeorology.

[103] Kubota T (2009). Verification of high-resolution satellite-based rainfall estimates around Japan using a gauge-calibrated ground-radar dataset. Journal of the Meteorological Society of Japan. Ser. II.

[104] Tian, Y. *et al*. Component analysis of errors in satellite-based precipitation estimates. *Journal of Geophysical Research: Atmospheres* **114** (2009).

[105] Derin, Y. *et al*. Evaluation of GPM-era global satellite precipitation products over multiple complex terrain regions. *Remote Sensing* **11** (2019).

[106] Donohue RJ, Roderick ML, McVicar TR (2012). Roots, storms and soil pores: Incorporating key ecohydrological processes into Budyko’s hydrological model. Journal of Hydrology.

[107] Liu Q (2016). The hydrological effects of varying vegetation characteristics in a temperate water-limited basin: Development of the dynamic Budyko-Choudhury-Porporato (dBCP) model. Journal of Hydrology.

[108] Kuligowski, R. J. An overview of National Weather Service quantitative precipitation estimates. United States, National Weather Service, Techniques Development Laboratory. https://repository.library.noaa.gov/view/noaa/6879 (1997).

[109] Wolff DB, Fisher BL (2008). Comparisons of instantaneous TRMM ground validation and satellite rain-rate estimates at different spatial scales. Journal of Applied Meteorology and Climatology.

[110] Beck HE (2020). Bias correction of global precipitation climatologies using discharge observations from 9372 catchments. Journal of Climate.

[111] Osborn TJ, Hulme M (1997). Development of a relationship between station and grid-box rainday frequencies for climate model evaluation. Journal of Climate.

[112] Pietersen JPJ, Gericke OJ, Smithers JC, Woyessa YE (2015). Review of current methods for estimating areal reduction factors applied to South African design point rainfall and preliminary identification of new methods. Journal of the South African Institution of Civil Engineering.

[113] Pedregosa F (2011). Scikit-learn: machine learning in Python. J. Mach. Learn. Res..

[114] Wilson AM, Jetz W (2016). Remotely sensed high-resolution global cloud dynamics for predicting ecosystem and biodiversity distributions. Plos Biology.

[115] Yamazaki D (2017). A high-accuracy map of global terrain elevations. Geophysical Research Letters.

[116] Legates DR, Bogart TA (2009). Estimating the proportion of monthly precipitation that falls in solid form. Journal of Hydrometeorology.

