# Supplementary material for: PPDIST, global 0.1° daily and 3-hourly precipitation probability distribution climatologies for 1979–2018
Source: Sci Data. 2020 Sep 11;7:302. doi: 10.1038/s41597-020-00631-x (PMC7486373; doi:10.1038/s41597-020-00631-x)
Supplement: Supplementary file 1 — Supplementary Information [file 41597_2020_631_MOESM1_ESM.pdf]

Supplement of “PPDIST: global 0.1° daily and 3-hourly precipitation probability distribution climatologies for 1979–2018”

July 7, 2020

List of Figures

S1 Record lengths of the (a) daily and (b) 3-hourly gauge data. 2

S2 The > 0.1 mm 3h^{-1} P occurrence according to (a) the gauge observations, (b) the PPDIST dataset, (c) the ERA5 reanalysis, and (d) the IMERG satellite product. The other PPDIST P occurrence indices can be viewed by accessing the dataset. 3

S3 The 15-year return-period 3-hourly P intensity according to (a) the gauge observations, (b) the PPDIST dataset, (c) the ERA5 reanalysis, and (d) the IMERG satellite product. IMERG has data gaps at high latitudes ($>$ 60°N/S) precluding the calculation of the 15-year return-period 3-hourly P intensity. The other PPDIST P intensity indices can be viewed by accessing the dataset. 4

S4 Performance of the PPDIST dataset, the ERA5 reanalysis, and the IMERG satellite product in estimating the 3-hourly P occurrence and peak P intensity indices. For the PDIST dataset, we calculated the mean of the ten validation scores (one for each cross-validation iteration). Training scores were not shown as they were nearly identical to the validation scores. All scores were calculated using square-root-transformed observed and estimated values. 5

S5 PPDIST uncertainty estimates for (a) the > 0.1 mm 3h^{-1} P occurrence and (b) the 15-year return-period 3-hourly P intensity. The uncertainty represents the spread of the five cross-validation iterations. 5

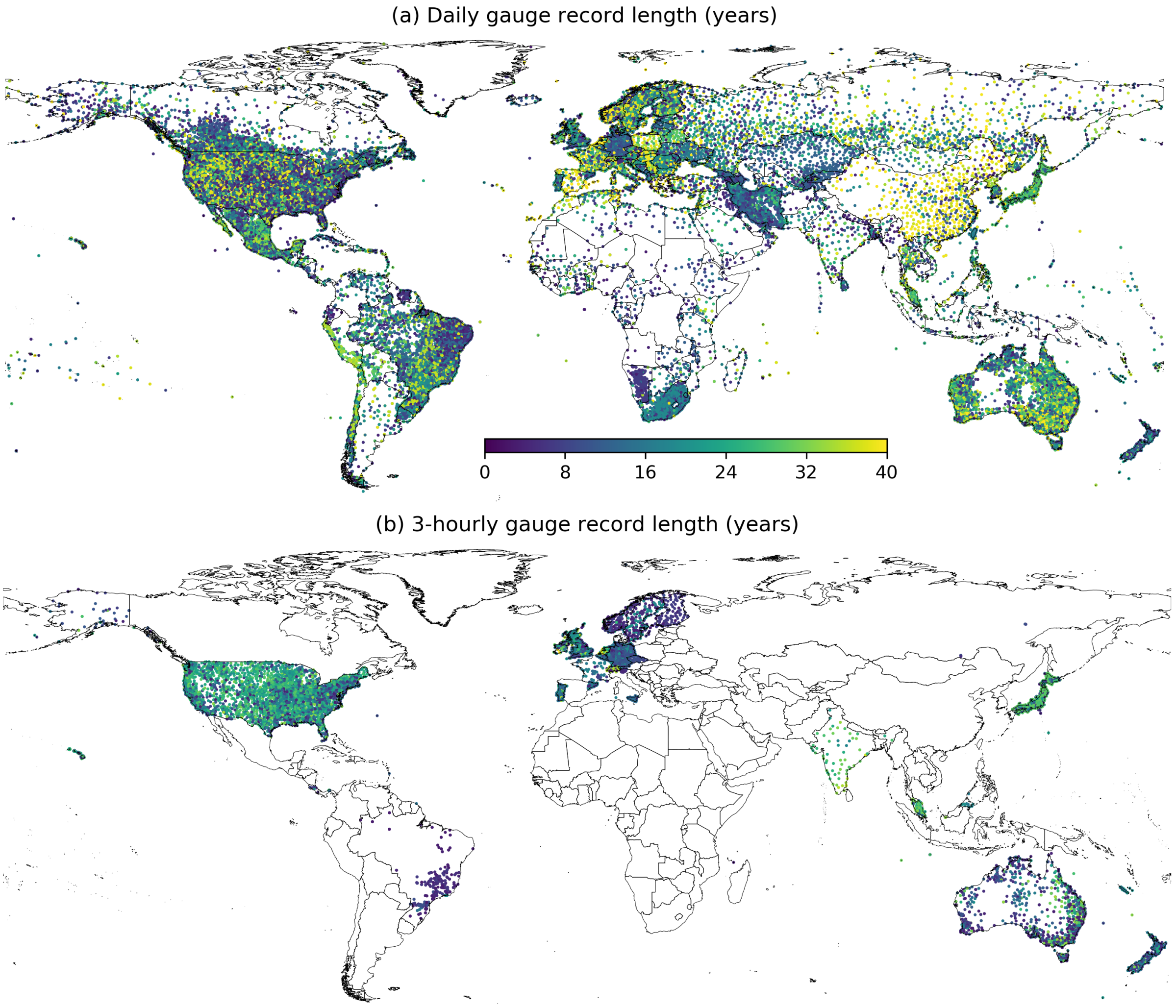

Figure S1: Record lengths of the (a) daily and (b) 3-hourly gauge data.

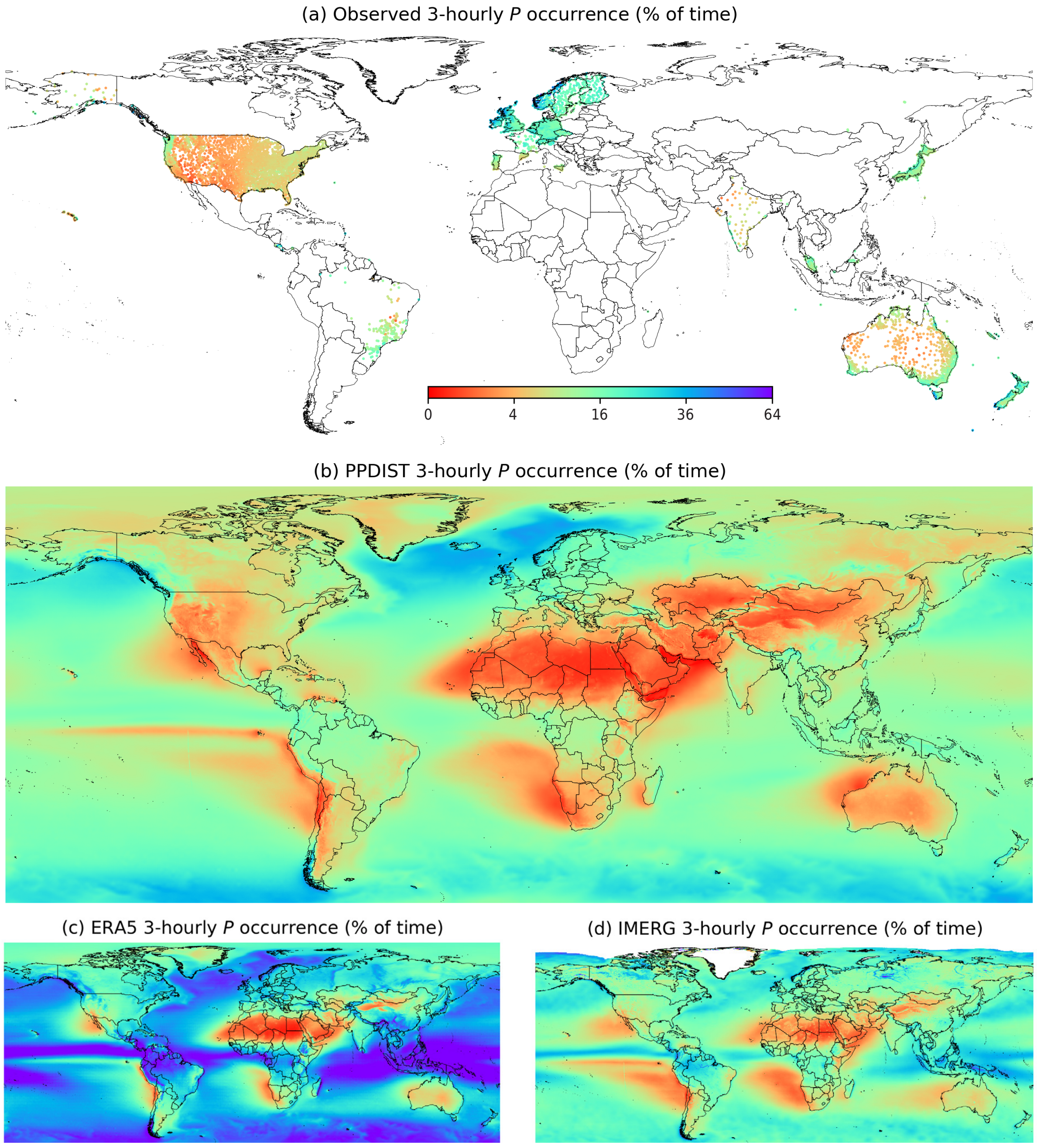

Figure S2: The > 0.1 mm 3h^{-1} P occurrence according to (a) the gauge observations, (b) the PPDIST dataset, (c) the ERA5 reanalysis, and (d) the IMERG satellite product. The other PPDIST P occurrence indices can be viewed by accessing the dataset.

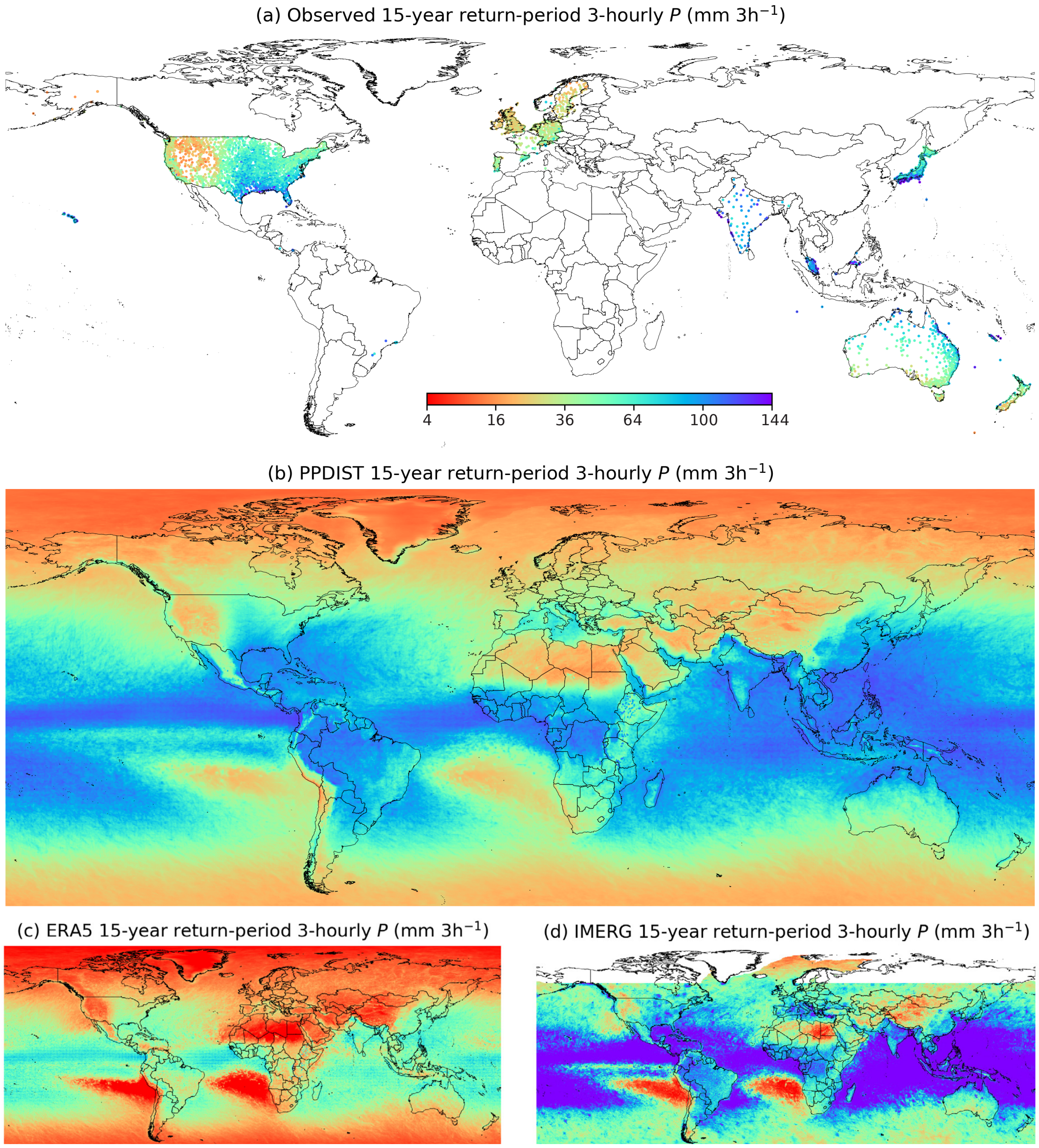

Figure S3: The 15-year return-period 3-hourly P intensity according to (a) the gauge observations, (b) the PPDIST dataset, (c) the ERA5 reanalysis, and (d) the IMERG satellite product. IMERG has data gaps at high latitudes ($>$ 60°N/S) precluding the calculation of the 15-year return-period 3-hourly P intensity. The other PPDIST P intensity indices can be viewed by accessing the dataset.

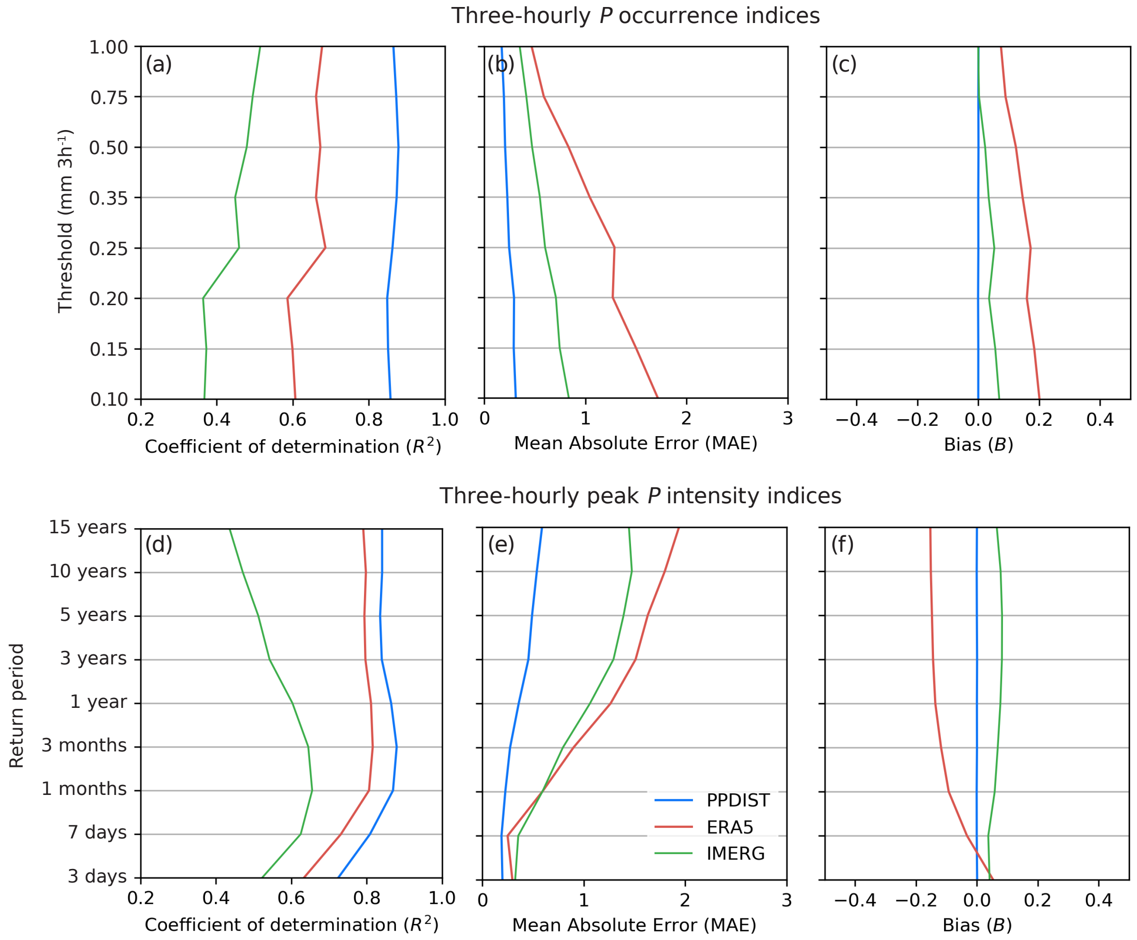

Figure S4: Performance of the PPDIST dataset, the ERA5 reanalysis, and the IMERG satellite product in estimating the 3-hourly P occurrence and peak P intensity indices. For the PDIST dataset, we calculated the mean of the ten validation scores (one for each cross-validation iteration). Training scores were not shown as they were nearly identical to the validation scores. All scores were calculated using square-root-transformed observed and estimated values.

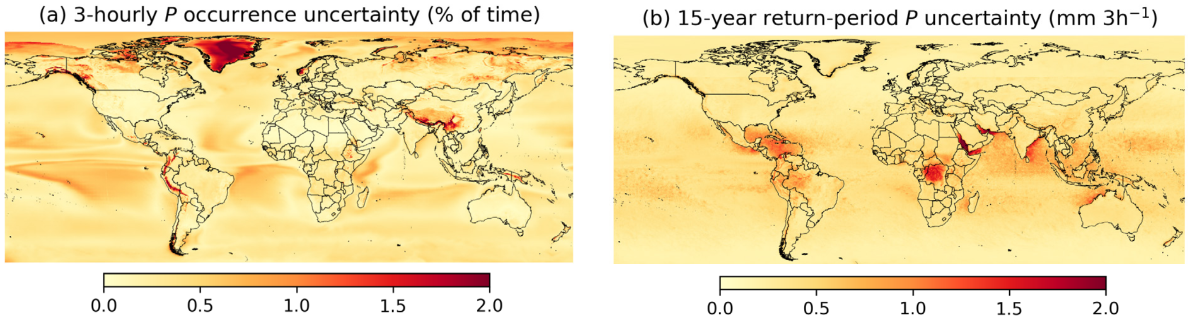

Figure S5: PPDIST uncertainty estimates for (a) the > 0.1 mm $3h^{-1}$ P occurrence and (b) the 15-year return-period 3-hourly P intensity. The uncertainty represents the spread of the five cross-validation iterations.
